# Quantitative Determination of Whey Protein to Casein Ratio in Infant Formula Milk Powder

**DOI:** 10.3389/fchem.2022.872251

**Published:** 2022-05-10

**Authors:** Tao Xu, Jingyao Chen, Kai Yang, Weicang Qiao, Junying Zhao, Lijun Chen

**Affiliations:** ^1^ School of Bioengineering, Dalian Polytechnic University, Dalian, China; ^2^ National Engineering Research Center of Dairy Health for Maternal and Child, Beijing Sanyuan Foods Co. Ltd, Beijing, China; ^3^ Beijing Engineering Research Center of Dairy, Beijing Sanyuan Foods Co. Ltd., Beijing, China; ^4^ South Asia Branch of National Engineering Center of Dairy for Maternal and Child Health, Guilin University of Technology, Guilin, China

**Keywords:** relative quantitative, mass spectrometry, infant formula, protein, peptides

## Abstract

This study was aimed to establish a method for quantitatively determining the ratio of whey protein in the total protein of infant formula by respectively selecting two characteristic peptides from whey protein and casein and calculating the ratio between the characteristic peptides. A nanoliter high-performance liquid chromatography tandem high-resolution mass spectrometry (Q Exactive) was used to simultaneously detect the characteristic peptides of two main whey proteins and two main caseins. The characteristic peptides were calculated, predicted, and screened using the ExPASy website, and peptide information was confirmed by database retrieval after the analysis by using a high-resolution mass spectrometer. The matrix effect was compensated by comparing the characteristic peptides in whey protein with those in casein protein, in which isotope internal standards were not required. The influence of the changes of the protein content in whey protein and casein on the detection method was eliminated by the calculation formula designed by ourselves. In this detection method, the sample was stable in the total protein concentration range of between 0.1 and 0.4 mg/ml. In the simulated industrial processing environment, with desalted whey powder, the recovery rate was 98.63–113.33% under different spiked levels with good reproducibility (RSD<8%). The RSDs of intraday and interday precisions were 2.03–9.35% and 0.61–11.02%, respectively. The different processing procedures of samples had no significant impact on the detection of whey protein (RSD% for milk samples treated by different processing techniques was 2.97%). The quantitation method of whey protein was applied to evaluate the whey protein content in different brands of commercially available milk powder. In summary, the proposed method was applicable for quantitative analysis of whey proteins in the infant formula.

## Introduction

As an important nutrient with high-nutritional value and rich active substances, whey protein is often added to various foods such as infant formula. The infant formula is used as a substitute for human milk for the newborns unavailable to adequate human milk, in which the content of whey protein is particularly important. It has been established that whey protein accounts for about 18% of the total protein content in cow milk, and it accounts for 60%–70% of the total protein content in human milk. To meet the nutritional needs of infants for growth and development, adequate whey protein is often added in the formula to make it closer to human milk. In the past few years, several commonly used methods for detecting whey protein in formula milk powder have been proposed and applied in practice, such as sodium dodecyl sulfate–polyacrylamide gel electrophoresis (SDS–PAGE), amino acid conversion algorithm, capillary electrophoresis (CE), high-performance liquid chromatography (HPLC), and liquid chromatography–mass spectrometry (LC–MS) (Kinghorn et al., 1995; Czerwenka et al., 2007; Bonfatti et al., 2013; Feng and Baugh, 2013).

The invention of polyacrylamide gel electrophoresis in 1959 ushered in a new era of modern electrophoresis. Nowadays, due to its simple operation, fast detection speed, and low cost, it is often used for the qualitative and quantitative detections of biological macromolecules (Restani et al., 1995; Aludatt et al., 2017). But when this method is used to detect whey protein in infant formula milk powder, the detected electrophoresis band will change, thus affecting the detection result, which might be caused by the complicated process under high temperature and high pressure in the production process of the infant formula (Lemos et al., 2015; Krause and Goldring, 2019). As one of the most commonly used detection methods, HPLC separates and analyzes the components in the mixture according to their difference in retention time. Modern capillary electrophoresis was invented by Jorgenson and Lukacs in 1981. Due to its high selectivity and superior separation effect of proteins, it has been widely used in protein analysis (Veledo et al., 2005; Grochocki et al., 2015), but when it was used to separate the proteins in formula milk powder with complex matrices, adhesion occurred in the related protein peak. In the latest ISO standard (ISO 23293-2020), the sub-protein peaks in whey protein are compared with casein as a whole, thus a more accurate capillary electrophoresis method can be obtained for the detection of whey protein in formula milk powder (Feng et al., 2018). In 2013, Wesley proposed to quantify whey protein in formula milk powder with the amino acid model, so as to avoid the influence of protein structure changes on the detection of whey protein, and to quantify whey protein more accurately from the amino acid level (Feng and Baugh, 2013). However, because amino acids are present in each protein, the conversion of the amino acid amount to the whey protein content can only be completed by summarizing experimental experience. On the other hand, the incorporation of other miscellaneous proteins will cause great changes in the test results (Feng and Baugh, 2013). At the end of the 1980s, John Fenn invented electrospray electrolysis and created a new era of proteomics analysis. The extremely sensitive and selective ability of mixture analysis was obtained using the combination of physical separation capabilities of LC and universal mass analysis capabilities of MS (Fenn et al., 2012; Ke et al., 2017; Wang et al., 2018). The absolute quantification of whey protein in formula milk powder can be achieved using the internal standard method of isotope peptides. However, it has been found in recent years that isotope-labeled peptides and characteristic peptides may behave differently in the pretreatment process, and some peptides and the peptides labeled with isotope might mutually interfere with each other, causing distortion of the mass spectrum signal (Qian et al., 2006; Higgs et al., 2008; Chen and Pramanik, 2009; Winter et al., 2009).

To precisely quantify whey protein in formula milk powder, the influence of the denaturation behavior of whey protein during processing on the detection result should be prevented. This denaturation behavior can further amplify the error of some protein detection methods (SDS–PAGE, CE, and HPLC). The influence of protein denaturation can be effectively prevented using the method of protein quantification at the levels of amino acid and peptide. On the other hand, the unpracticability of the method limits the application of the detection method in the production process. The high price of synthetic internal standards for LC–MC detection greatly increases the detection costs. However, when internal standards are not used, the difference in the matrix effect and ionization efficiency leads to great error in the detection results.

This study detected the labeled isotope with HC–MC and made corrections according to other peptides in the sample without using the isotope internal standard, determined the whey protein content according to the relationship between the ratio of whey protein to casein and the peak area ratio of the respective characteristic peptides, and eliminated the difference between the matrix effect and ionization efficiency.

## Materials and Methods

### Materials

Nanoliter high-performance liquid chromatography UltiMate 3000 RSLCnano (Thermo Fisher Scientific Company) was used in this study.

Q Exactive-combined quadrupole Orbitrap mass spectrometer (Thermo Fisher Scientific Company) was used in this study.

The chemicals and reagents used in this study were purchased from Sigma-Aldrich, including the standard protein product with the purity of 85% α-lactalbumin, 90% *β*-lactoglobulin, 70% *α*-casein, 98% *β*-casein, and alkaline bovine trypsin (Sigma, ≥10,000 N-benzoyl-l-arginine-ethyl ester, BAEE). The polyethersulfone fiber membrane was purchased from Agela and Phenomenex (Article No. AS051320-19). Mass spectrometry analysis software: Thermo Proteome Discoverer (version 1.4) from Thermo Fisher Scientific Company; Xcalibur (Thermo Fisher Scientific Company) ingredient list for desalting whey powder. A commercial infant formula powder has been used. Desalted whey powder from the model D90 was used (protein 14.01%, lactose 83.69%, and moisture 1%, Fonterra Company, New Zealand).

## Methods

### Enzymolysis Condition

The enzymolysis efficiency of whey protein and casein was different, which was related to the enzyme content and enzymolysis time. To reduce the detection error, it is very important to ensure the stability of the peptide obtained by protease hydrolysis. The change of peptide ratio of whey protein and casein in formula milk powder with enzymatic hydrolysis time was detected. Experiments were performed using formulated milk powder as the substrate and an enzymatic digestion using bovine-derived alkaline trypsin, where samples were configured using formulated milk powder at a protein concentration of 0.2 mg/ml (see pretreatment conditions for detailed procedure). The optimal enzymatic hydrolysis conditions were finally determined to be as follows: enzyme concentration:substrate concentration of 1:40; lysed for 28 h.

### Pretreatment Conditions

Formula milk powder was dissolved in ultrapure water so that the protein concentration was between 0.1 mg/ml and 0.4 mg/ml, 250 μL solution was taken into the centrifuge tube, 150 μl of 500 mmol/L ammonium bicarbonate and 10 μl of 500 mmol/L dithiothreitol were added for reduction in the water bath at 70°C for 30 min. After cooling, 30 μl of 500 mmol/L iodoacetamide was added for alkylation in the dark for 30 min. After the aforementioned mixture was exposed to light for 10 min (to decompose iodoacetamide), 10 μl of 100 mmol/L calcium chloride solution and 50 μl of 0.5 mg/ml trypsin solution were added for enzyme digestion at 37°C for more than 28 h. After 10 μL of formic acid solution was added, the aforementioned mixture was set aside for 15 min to terminate the digestion. Pure water (490 μL) was added into the centrifuge tube and mixed well. After filtration with a 0.22 μm polyethersulfone fiber membrane, nanoliter liquid chromatography was applied for selective ion scanning analysis.

### Preparation of Mixed Standards

Four protein standards of α-lactalbumin, *β*-lactoglobulin, α-casein, and *β*-casein were used to prepare mixed protein standards. For the milk produced by cows of different breeds in different seasons and regions, the contents of protein components in milk are different, resulting in changes in the contents of protein components in whey protein and casein protein. In this study, by referring to relevant literature (Kinghorn et al., 1995; Lönnerdal, 2014) and considering the actual processing loss and the error of detection method, the protein content of each component was set as follows: α-lactalbumin and *β*-lactoglobulin accounted for 20% and 50% of whey protein, respectively, and α-casein and *β*-casein accounted for 50 and 40% of casein, respectively (Tegge and Strke, 1982; Kunz and Lönnerdal, 1992; Kinghorn et al., 1995; Lara-Villoslada et al., 2005). According to the proportion of each protein component in whey protein and casein, the whey protein standard solution (20% *α*-lactalbumin and 50% *β*-lactoglobulin) and the casein standard solution (50% *α*-casein and 40% *β*-casein) were prepared. The prepared whey protein standard solution had the same protein concentration with that of the casein standard solution.

### Liquid Chromatographic Conditions

Acclaim® PepMap RSLC (75 μm × 15 cm, nanoViper C18, 2 μm, 100A Thermo Fisher Scientific) was selected as the separation column. Acclaim PepMapTM 100 (75 μm × 2 cm, nanoViper C18, 3 μm, 100A Thermo Fisher Scientific) was chosen as the enrichment column. The aqueous solution of 0.1% formic acid and 2% acetonitrile was taken as the mobile phase A; the solution of 0.1% formic acid acetonitrile and 20% water as the mobile phase B. The column temperature of 50°C, the flow rate of 0.25 μl/min, and the injection volume of 1 μl were adopted as the chromatographic parameters. The sample was enriched on the enriching column with the aqueous solution of 2% acetonitrile at the flow rate of 0.3 μl/min. The elution procedure was as follows. (Table 1).

**TABLE 1:** Gradient elution conditions.

(Time, min)	0 (%)	5 (%)	65 (%)	70 (%)	75 (%)	76 (%)
A	96	96	78	10	96	96
B	4	4	22	90	4	4

### Mass Detection Conditions

The Q Exactive-combined quadrupole Orbitrap mass spectrometer equipped with the Nanospray Flex nanoliter electrospray source was applied, in which the full mass was used to search for characteristic peptides, and parallel reaction monitoring (PRM) mode was used for quantitative detection of characteristic peptides. The data collection (ESI^+^) mode of electrospray positive ion was adopted; Thermo Proteome Discoverer (version 1.4) was used to search the sequence database for analysis. The parameter settings of a relevant instrument and software (Xcalibur) were as follows: full scan mode was adopted for the data-dependent acquisition mode; Orbitrap acquisition was used for the primary mass spectrometer with the scanning range of 200–1,000 m/z and the resolution of 17500. MS1 was selected for the initial mass-to-charge ratio with the separation window of 1.0 m/z and the collision energy of NCE 27/28. The PRM mode was used as the quantitative scanning mode, and the parent ion information of characteristic peptide was set in the inclusion list.

### Analysis of Mass Spectrometric Data

The raw data files of the obtained samples were compared with the database using Thermo Proteome Discoverer 1.4 software. SEQUEST HT was used as the search software of the comparison database, and the database was derived from UniProt (*Homo sapiens* and *Bos taurus* were used for the human and cattle, respectively). The specific parameters were set as follows: enzyme of trypsin (full), maximum missed cleavage site of 2, parent ion mass deviation of 10 ppm, and daughter ion mass deviation of 0.02Da. Dynamic modification was set as oxidation/M +15.995 Da and deamidation/N, Q +0.984Da, and fixed modification was set as carbamidomethyl/C +57.021Da.

### The Calculation Formula

The final test results are not only affected by the changes in the protein content of each composition in whey protein and casein during the production process but also by the differences in the raw materials and the nutrients supplemented by the manufacturer. In order to eliminate the errors caused by the changes of the protein content, the ratios of two groups of proteins were chosen in the experiment, which were further corrected by formula calculation.

The main proteins in whey protein and casein in each group were compared. The ratios of the two groups of peptides were TP:FAL (*β*-Lg:*α*-cs) and LD:VL (*α*-La:*β*-cs) (TP, LD, FAL, and VL are the amino acids at the beginning of each peptide. TP: threonine and proline; FAL: phenylalanine, alanine, and leucine; LD: leucine and aspartic acid; VL: valine and leucine. They are used here to represent each peptide; the two peptides from whey protein were randomly compared with the two peptides from casein), respectively. According to the standard curve of the detected peptide response area ratio and protein ratio, two ratios of whey protein to casein of M (TP:FAL) and N (LD:VL) were obtained. The ratios of M and N can be changed into the fraction form as shown in the following Eqs 1, 2:

$$M=\frac{m1}{m2}$$ (1)

$$N=\frac{n1}{n2}.$$ (2)

The ratios of the two groups of peptides were obtained from one analysis in the same matrix. It was specified that m1+m2 = n1+n2 = 1, and postulated that the protein concentration was X. Since the standard curve is drawn by mixing the standard protein solutions, the four main proteins of *β*-lg, *α*-la, *α*-cs, and *β*-cs in the mixed standard solution are shown in the following formulas:

Whey protein = 20% *α*-la + 50% *β*-lg + 30% miscellaneous protein.

Casein = 50% *α*-cs + 40% β-cs + 10% miscellaneous protein.

The true value of the four proteins is obtained in the following Eqs 3–6:

$$\text{β}-1\text{g}=\frac{m1}{m1+m2}X*50\%,$$ (3)

$$\text{α}-\text{c}\text{s}=\frac{m2}{m1+m2}X*50\%,$$ (4)

$$\text{α}-1\text{a}=\frac{n1}{n1+n2}X*20\%,$$ (5)

$$\text{β}-\text{c}\text{s}=\frac{n2}{n1+n2}X*40\%.$$ (6)

According to the proportion of each protein in the corresponding protein, the actual ratio of whey protein:casein is obtained by the following Eq. 7:

$$\frac{\frac{n1}{n1+n2}X*20\%+\frac{m1}{m1+m2}X*50\%}{0.7}/\frac{\frac{m2}{m1+m2}X*50\%+\frac{n2}{n1+n2}X*40\%}{0.9}=\frac{18n1+45m1}{35m2+28n2}=\frac{63MN+18N+45M}{35N+28M+63}.$$ (7)

The percentage of whey protein in total protein is obtained by the following Eq. 8:

$$\frac{63MN+18N+45M}{63MN+53N+73M+63}\times 100\%,$$ (8)

where M and N are the ratios of whey protein to casein determined by the two peptides of TP:FAL and LD:VL, respectively.

## Results and Discussion

### Choice of Protein and Characteristic Peptides

**FIGURE 1:**
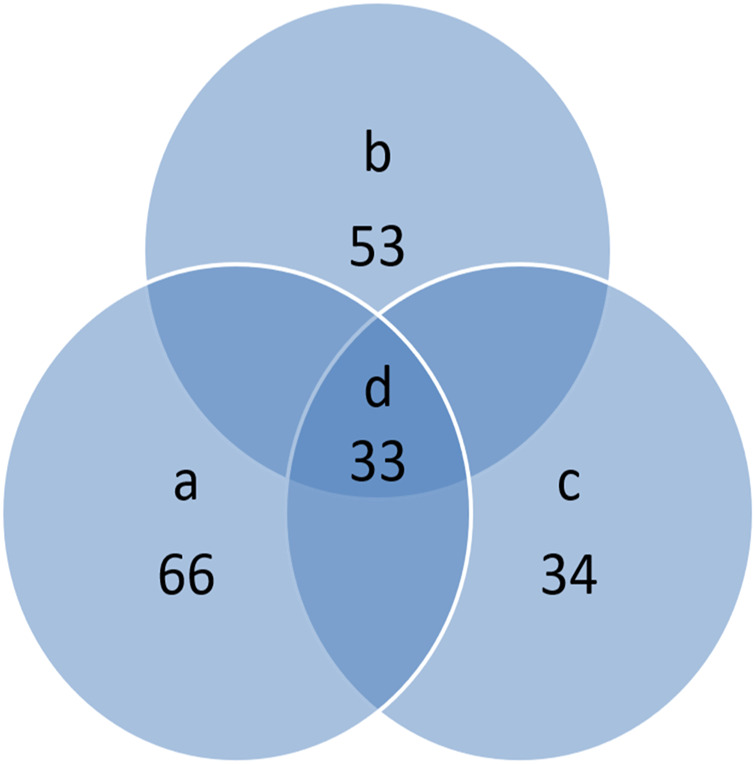
Comparison of actual number of detected peptides with theoretical peptides.

To bring the protein content of milk formula closer to that of human milk, a certain amount of milk protein is industrially added to meet the criterion of whey protein:casein ratio of 60:40 in the infant formula (Kunz and Lönnerdal, 1992; Wood et al., 2021). Milk protein is composed of whey protein and casein, in which α-lactalbumin and *β*-lactoglobulin account for 70–80% of whey protein (Lara-Villoslada et al., 2005; Lönnerdal, 2014) and α-casein and *β*-casein account for about 90% of casein (Kunz and Lönnerdal, 1992). Therefore, the two main types of whey protein and two main types of casein were used to detect the ratio of whey protein to casein in the infant formula. Four cow protein standard substances and commercially available formula milk powder were chosen to perform a full scan of peptides and simulate enzyme digestion of all the aforementioned protein with the Peptide Mass tool provided by the ExPASy website (https://web.expasy.org/peptide_mass/) to get the theoretically calculated peptides and screen the specific peptides of target protein (Figure 1). As shown in Figure 1 , the letter a represents the theoretical digestion peptides; the letter b represents the whey protein peptides detected after the enzyme digestion of formula milk powder; the letter c represents the peptides detected after standard protease digestion; the letter d represents the common peptides detected in all three samples. To ensure the representativeness of the selected peptides, BLAST comparison analysis of proteins was performed on the NCBI website (https://www.ncbi.nlm.nih.gov/). Considering that the difference in peptide length might have an impact on mass spectrometry detection, peptide sequences are selected with similar lengths as much as possible, and finally the characteristic peptides of four kinds of proteins are selected (Table 2).

**TABLE 2:** Ion information of characteristic peptides.

Protein	Peptide	Mass (KDa)	Daughter ion (KDa)	Time (Min)
*β*-lg	TPEVDDEALEK	623.29	199.10	27.5
*α*-la	LDQWLCEK	546.23	268.16	55.2
*α*-cs	FALPQYLK	490.19	120.08	57.8
*β*-cs	VLPVPQK	390.75	372.22	23.7

## Method Validation

### Peptide Specificity

**FIGURE 2:**
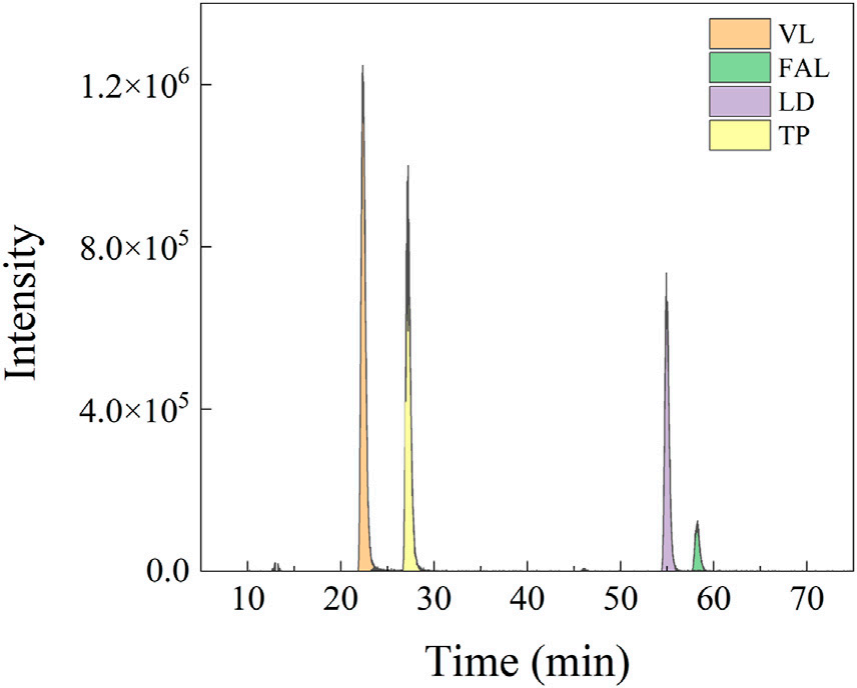
Quantitative ion peak shape of four peptides.

**FIGURE 3:**
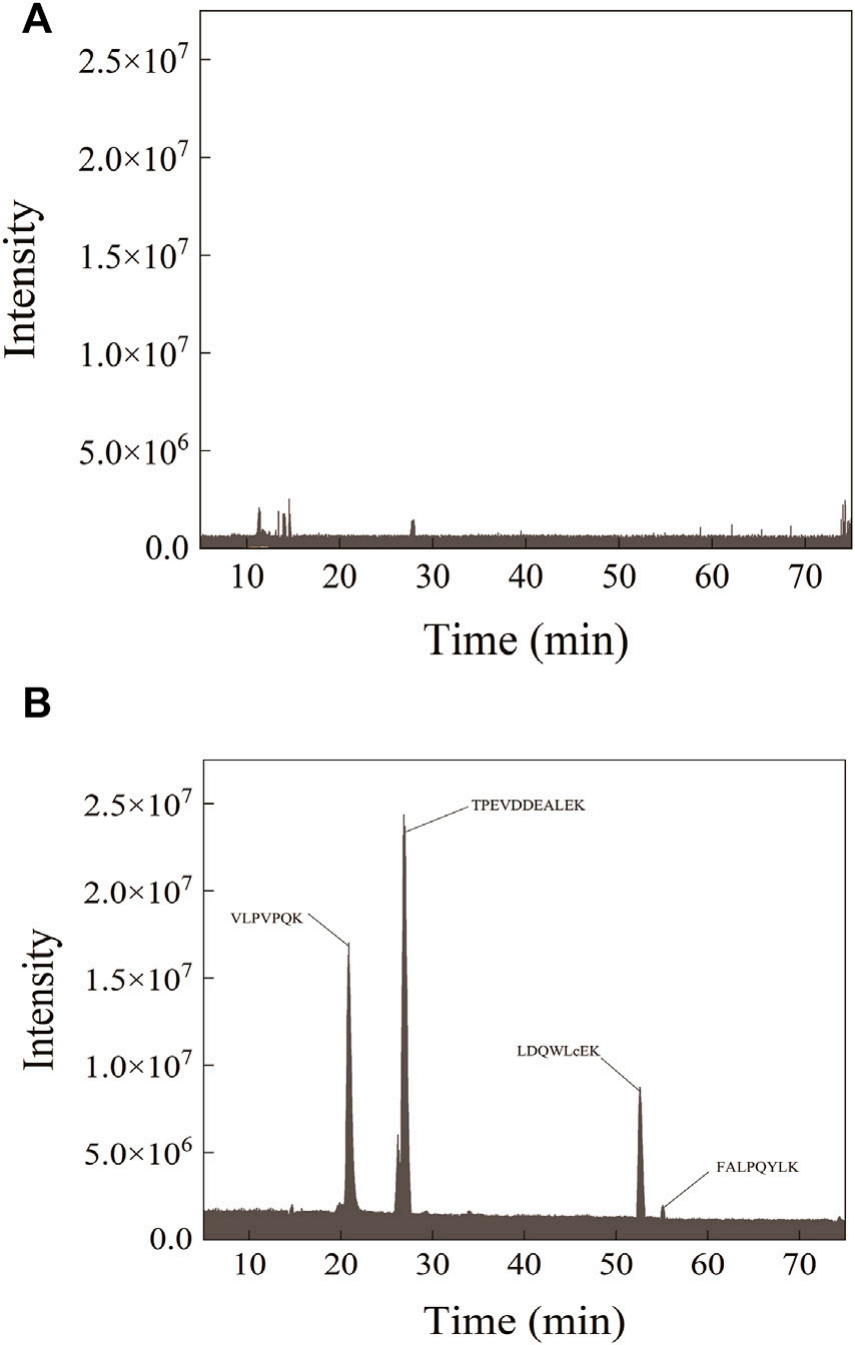
Peptide ion diagrams of mass spectrometry PRM before **(A)** and after **(B)** enzymatic digestion of the sample.

In the detected sample, the parent ions of the four characteristic peptides and the selected single-daughter ions showed steep and symmetrical peaks without interference (Figure 2). Separation of four peptides from infant formula milk powder was detected by mass spectrometry, with the protein concentration 0.2 mg/ml. There was no target peak in the chromatogram of the milk powder sample undigested by trypsin (Figure 3).

### Complete Enzymolysis

**FIGURE 4:**
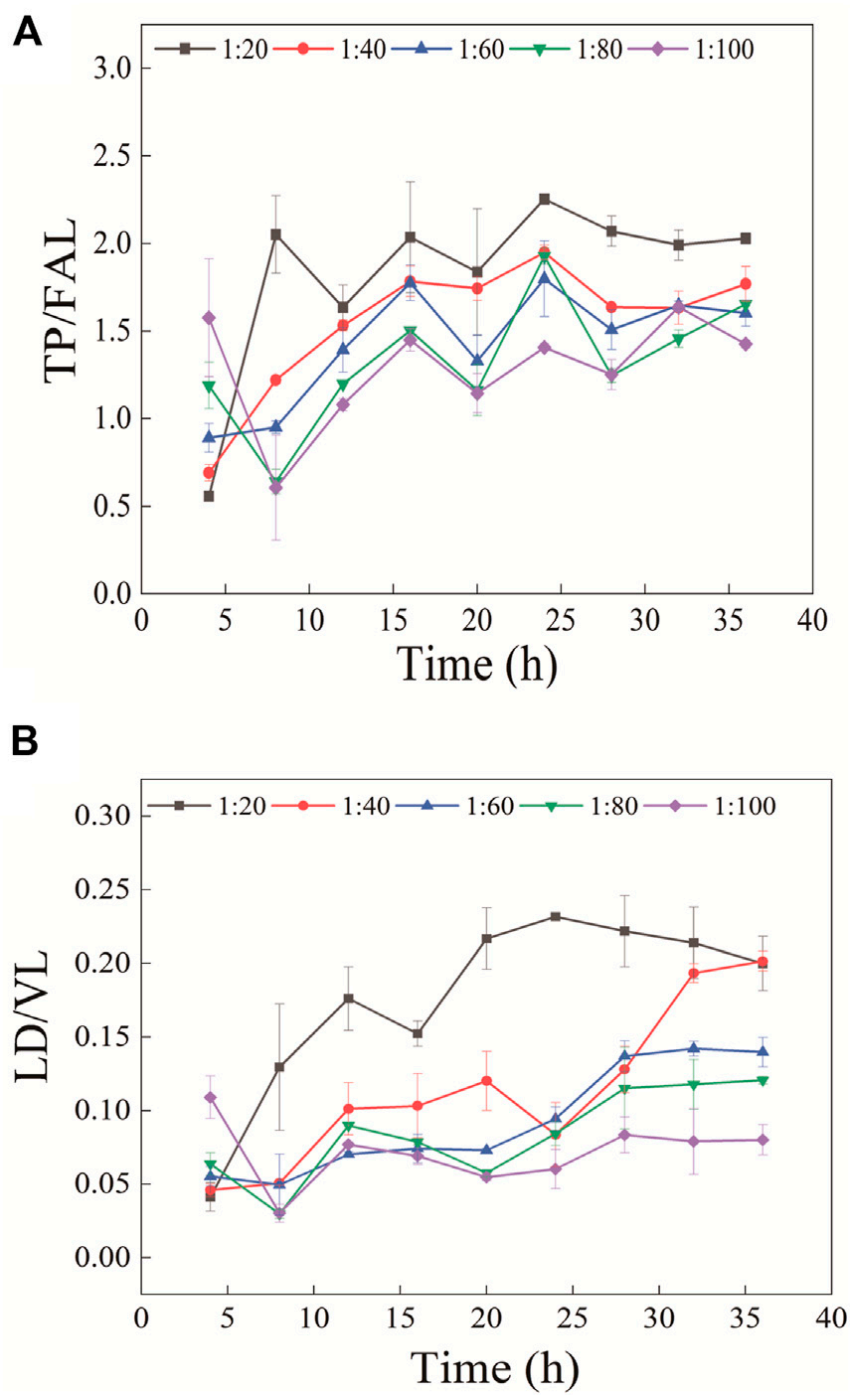
Ratio of peptides **(A)** TP/FAL and **(B)** LD/VL changes with enzyme concentration and enzymatic hydrolysis time (*n* = 3).

In the experiment, the enzymolysis conditions for stable protein cleavage were obtained by changing enzymolysis time and enzyme concentration. The finally balanced peptide ratios obtained at different enzyme concentrations were different (Figure 4). This might be caused by the enzyme inactivation due to autohydrolysis during the enzymolysis process, which made it impossible to further enzymatically hydrolyze protein. Therefore, the ratio of peptides after stable enzymatic hydrolysis changes in a certain trend with the enzyme concentration. The final peptide ratio was consistent in the TP/FAL digestion curve. Compared with TP/FAL, this phenomenon was more evident in LD/VL. In the LD/VL enzymatic hydrolysis curve, the final peptide ratio of the enzyme concentration tended to be consistent when the enzyme concentration was greater than 1:40. Therefore, we believe that the protein in this group can be completely digested into peptides at the enzyme concentration of 1:40. It was shown that with the concentration ratio of enzyme and substrate of 1:20, the ratio of peptides in the two groups tended to be stable after 28 h, and the error range was smaller.

### Linearity Verification

**FIGURE 5:**
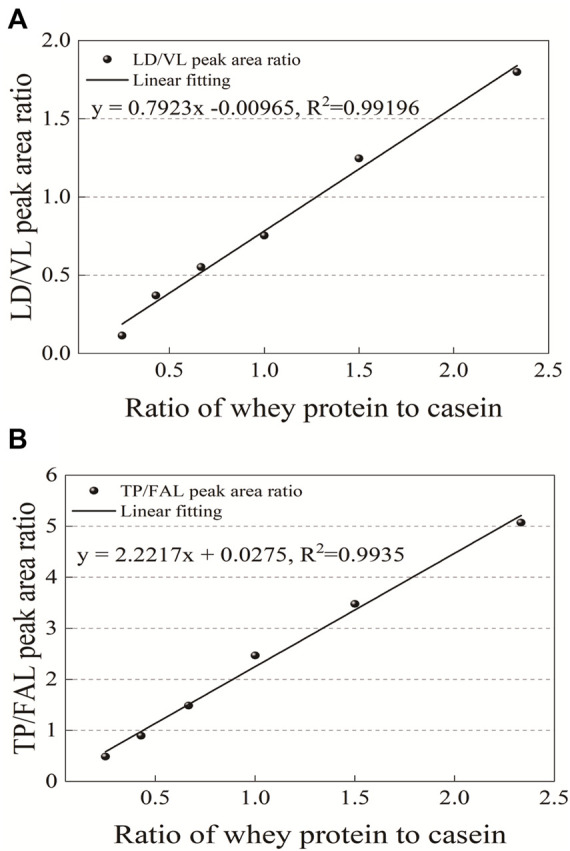
Standard curve of the ratio of peptides in **(A)** LD/VL and **(B)** TP/FAL.

The relationship between the ratios of the two peptides was observed according to the gradient of six different concentration ratios of whey protein to casein (20:80, 30:70, 40:60, 50:50, 60:40, and 70:30) in the standard solution prepared with different ratios of whey protein and casein. Using this as a standard, the mass ratio of the two proteins in the sample can be obtained from the peak area ratio of peptides in the two groups after enzymatic hydrolysis (Figure 5). The 2-level linear regression equations of the characteristic peptides had a good linear relationship and a determination coefficient (*r* ^2^ > 0.99) (Table 3).

**TABLE 3:** Linear regression equation of the ratio of peptides in the two groups (*n* = 3).

	Linear regression equation	*R* ^2^	RSD (%)
TP/FAL	y = 2.2217x + 0.0275	0.9935	2.88–8.06
LD/VL	y = 0.7923x-0.0096	0.992	3.01–9.75

### The Concentration Range for the Method

**FIGURE 6:**
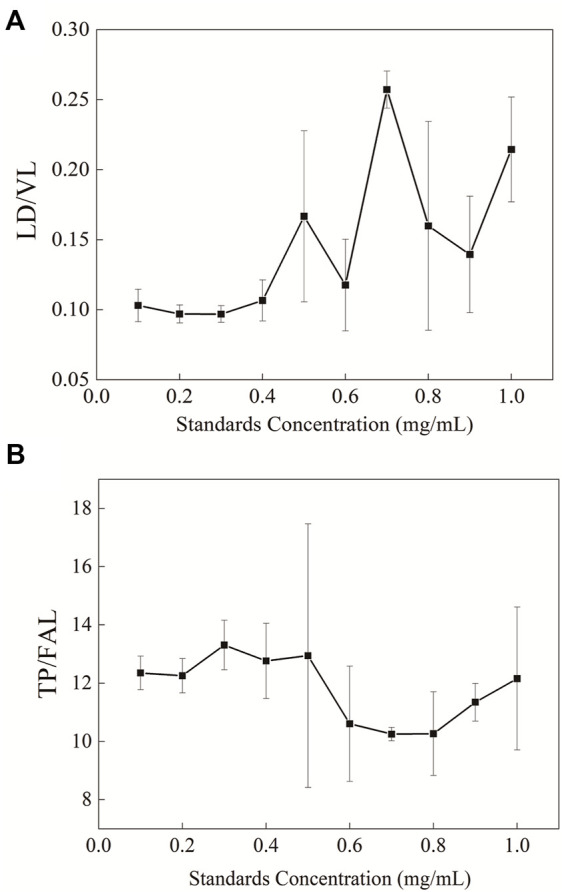
Changes in the ratio of peptide in the samples with different protein concentrations after enzymolysis **(A)** LD/VL and **(B)** TP/FAL.

Whey protein was quantified with peptide ratios in four different proteins in the study. In order to ensure the stability of enzymatic hydrolysis under the experimental conditions, the sample detection range of this method was determined. The changes in the ratio of peptides in two groups after the aforementioned pretreatment were observed using 10 different protein concentration gradients (Figure 6). It was shown that when the protein concentration in the sample was below 0.4 mg/ml, the ratio of peptides in the two groups remained stable with small variation in the error.

### Verification of the Recovery Rate

**FIGURE 7:**
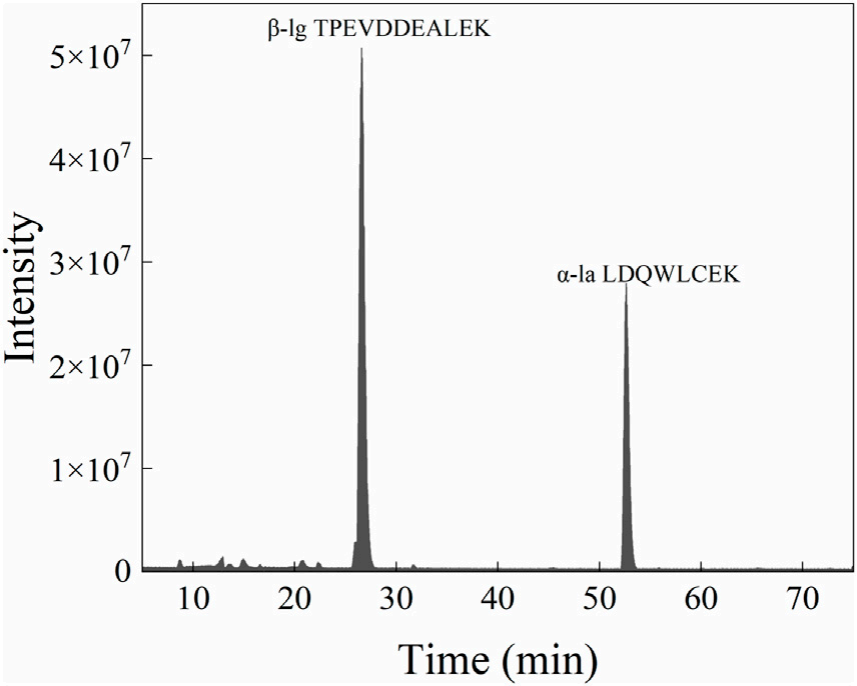
Mass spectrometric analysis results of desalted whey powder with protein concentration of 0.2 mg/ml.

To simulate the change of whey protein in the industrial production process, the demineralized whey powder that was used to offer whey protein in the production process of formula milk powder (Figure 7) (it was determined that the protein in the demineralized whey powder did not contain casein, and the content of whey protein accounted for 14% in the total whey powder) was used as the spiked standard, and the spiked samples were pretreated and analyzed by the aforementioned method. The recovery test was performed by comparing the measured concentration of the control sample and the spiked sample with the theoretical concentration (Table 4). The recovery rate of the spiked sample was between 98.63% and 113.33%, and the RSD was between 0.84% and 7.42%, which indicated that the method had good accuracy.

**TABLE 4:** Spiked recovery rate (*n* = 3).

	Spiked level (mg)	Theoretical value (mg)	Detection value (mg)	Recovery (%)	RSD (%)
Substrate	0	—	19.48	—	7.35
Desalted whey powder	30	23.08	23.03	98.63	0.84
	60	26.68	26.90	103.06	7.42
	90	30.28	31.72	113.33	1.83
	120	33.88	34.11	101.62	0.84

### Intraday and Daytime Precision

According to the aforementioned experimental operation, three different commercial formula milk powders were pretreated in three consecutive days to evaluate the intraday and interday accuracies of the method. The whey protein content of the products varied according to the manufacturers of commercially available milk powder, and the TP/FAL value in sample three was higher than that in the other two samples (Table 5). The RSDs of intraday and interday precisions were 2.03–9.35% and 0.61–11.02%, respectively. It was shown that the method had good intragroup and intergroup precisions.

**TABLE 5:** Intragroup and intergroup precisions of formula milk powder (*n* = 3).

Sample	Peptide	Day	Intraday			Interday		
			Average	SD	RSD (%)	Average	SD	RSD (%)
1	TP/FAL	1	6.10	0.36	5.83	6.07	0.037	0.61
		2	6.03	0.29	4.88			
		3	6.09	0.16	2.61			
	LD/VL	1	0.59	0.022	3.69	0.60	0.014	2.28
		2	0.61	0.029	4.75			
		3	0.59	0.023	3.92			
2	TP/FAL	1	6.03	0.24	4.05	5.83	0.24	4.19
		2	5.60	0.17	3.05			
		3	5.62	0.13	2.24			
	LD/VL	1	0.59	0.031	5.26	0.61	0.032	5.29
		2	0.63	0.013	2.03			
		3	0.56	0.022	3.95			
3	TP/FAL	1	7.39	0.22	3.01	7.69	0.30	3.93
		2	7.69	0.16	2.07			
		3	7.99	0.66	8.32			
	LD/VL	1	0.54	0.045	8.29	0.60	0.066	11.02
		2	0.58	0.032	5.55			
		3	0.67	0.063	9.35			

Considering the purity issues of the experimentally used protein standards and the possible errors during experimental procedures, we considered the detection precision when the RSD of repeated detection was less than 10%. When verifying the interday precision, the samples to be tested are prone to change on their own due to the large time span, so we considered the precision of the assay to be qualified when the RSD was less than 12% (Ke et al., 2017).

### The Impact of Production and Processing on Testing Methods

The processing process of formula milk powder will affect the detection of whey protein, leading to changes in the content of whey protein in the same batch of samples before and after processing, so it was explored whether the production process might have an impact on this method. The changes of the whey protein content in milk powder were measured using the processes of sterilization (here, including pasteurization and ultrahigh-temperature sterilization), spray drying, and so on during the production of formula milk powder with raw milk as the starting material, so as to study the impact of processing technology on the detection method (Table 6). It was shown that the content of the finally detected whey protein was similar after the treatment using different processes, with an RSD of 2.97%, and the average value was almost the same as that of raw milk. This proves that processing technology in the industrial production process has no significant influence on the detection of the whey protein content by this method.

**TABLE 6:** Quantitative detection of whey protein with different processing techniques (*n* = 3).

	TP/FAL	LD/VL	Whey protein content (%)
Raw milk	0.42	0.0021	17.23
Homogenization	0.42	0.0021	17.39
Pasteurization	0.44	0.0020	17.93
Ultrahigh-temperature sterilization	0.45	0.0023	18.14
Spray drying	0.39	0.0024	16.59
Average value	0.42	0.0022	17.51
SD	0.024	0.00016	0.052
RSD (%)	5.65	7.50	2.97

### Application of the Method in Actual Sample Detection

Whey protein was detected in the infant formula of 11 domestic and international brands in this trial. The results showed that whey protein accounted for more than 60% of the total protein content in 81.8% of the formula milk powder, it was also very close to the 60% content standard in the remaining ones. (Table 7).

**TABLE 7:** Whey protein content of different brands of domestic and international formula milk powders.

Milk powder	TP/FAL	LD/VL	Whey protein content (%)
Sample 1	7.49	0.58	63.71
Sample 2	5.82	0.57	60.86
Sample 3	5.85	0.61	61.40
Sample 4	4.97	0.56	58.72
Sample 5	4.09	0.94	60.76
Sample 6	4.78	0.83	61.77
Sample 7	6.92	0.64	63.80
Sample 8	6.75	0.79	65.48
Sample 9	8.59	0.45	62.96
Sample 10	6.67	0.46	60.54
Sample 11	4.05	0.85	59.71

## Conclusion

This study established an analytical method to quantitatively determine the percentage of whey protein in the infant formula. By detecting the characteristic peptides of two whey proteins and two caseins, an ultrahigh-performance liquid chromatography–mass spectrometry (UHPLC–MS/MS) method was established for simultaneous quantification of two whey proteins and two caseins. In mass spectrometry analysis, matrix interference was avoided by comparing the two groups of peptides. The accuracy, sensitivity, and selectivity of the current method were verified by the calibration curve, intraday and interday precisions, and recovery rate (recovery rate 98.63–113.33%, determination coefficient *r* ^2^ > 0.99, intraday RSD <10%, and interday RSD <12%). A specific formula has been established to calculate the content of whey protein in formula milk powder. Through the detection of different processed samples, it was determined that the production process of formula milk powder would not change the testing results of this method. The method was applied to the routine testing of infant formulas of different commercial brands (*n* = 11). The results showed that the whey protein content accounted for more than 60% of the total protein in infant milk powders of most brands, and it was slightly less than 60% in only a small part of milk powder. Compared with other quantitative methods, this method is simple to operate in production practice, offers substantial savings in testing costs, and the test results are not affected by production and processing operations.

## Data Availability

The original contributions presented in the study are included in the article/Supplementary Material, further inquiries can be directed to the corresponding author.
